# Changes in the fecal bacterial microbiota associated with disease severity in alcoholic hepatitis patients

**DOI:** 10.1080/19490976.2020.1785251

**Published:** 2020-07-20

**Authors:** Sonja Lang, Bradley Fairfied, Bei Gao, Yi Duan, Xinlian Zhang, Derrick E. Fouts, Bernd Schnabl

**Affiliations:** a Department of Medicine, University of California San Diego, La Jolla, CA, USA; b Department of Medicine, VA San Diego Healthcare System, San Diego, CA, USA; c Division of Biostatistics and Bioinformatics, Department of Family Medicine and Public Health, University of California San Diego, La Jolla, CA, USA; d J.Craig Venter Institute, Department for Genomic Medicine, Rockville, MD, USA

**Keywords:** Microbiome, 16S sequencing, alcohol-related liver disease, metagenomics

## Abstract

**Background and Aims:**

Alcoholic hepatitis is the most severe form of alcohol-related liver disease. While the gut microbiome is known to play a role in disease development and progression, less is known about specific compositional changes of the gut bacterial microbiome associated with disease severity. Therefore, the aim of our study was to correlate gut microbiota features with disease severity in alcoholic hepatitis patients.

**Methods:**

We used 16S rRNA gene sequencing on fecal samples from 74 alcoholic hepatitis patients, which were enrolled at 9 centers in Europe, the United States, and Mexico in a multi-center observational study. The relative abundance of gut bacterial taxa on genus level, as well as the microbiome diversity, was correlated to various clinical, laboratory, and histologic parameters.

**Results:**

We observed a negative correlation between the model for end-stage liver disease score and Shannon diversity, independent of potentially confounding factors (P_adjust_ = 0.046). Alcoholic hepatitis patients with more severe disease had significantly decreased relative abundances of *Akkermansia* while the relative abundance of *Veillonella* was increased. We observed a reduction in the *Bacteroides* abundance (P_adjust_ = 0.048) and Shannon diversity (P_adjust_ = 0.018) in antibiotic-treated patients and patients receiving steroids had an increase in *Veillonella* abundance (P_adjust_ = 0.005), which was both independent of potentially confounding factors.

**Conclusion:**

We observed distinct changes in the gut bacterial microbiome of alcoholic hepatitis patients with more severe disease. The gut bacterial microbiome might be an attractive target to prevent and treat this deadly disease.

## Introduction

Alcohol-associated liver disease includes a wide spectrum of hepatic clinical syndromes and pathologic findings associated with heavy alcohol consumption^1^. Approximately 1 in 20 deaths worldwide is attributed to alcohol abuse, and alcohol-associated liver disease resulted in over 22,000 deaths in the US alone in 2017.^2^ Alcohol-associated liver disease includes steatosis, fibrosis, cirrhosis, and alcoholic hepatitis. These clinical entities, despite discrete definition, have substantial overlap and are closely interrelated. Simple steatosis involves fatty infiltration of the liver and is typically asymptomatic with normal or mild elevation in liver transaminases. A subset of these patients will go on to develop liver fibrosis, and ultimately, alcoholic cirrhosis. Alcoholic hepatitis is its own separate entity, which is related to but does not lie within the linear spectrum of steatosis, fibrosis, and cirrhosis in any predictable way. Thirty to 40% of chronic heavy drinkers will develop alcoholic hepatitis, but there is no clear identifiable trigger.^3^ Within alcoholic hepatitis, there is a wide range of disease severity, ranging from chronic and clinically silent to a fulminant syndrome of inflammation and cholestasis. Prognosis in alcoholic hepatitis varies widely, but is generally poor, with 30-day mortality reaching up to 50%.^4^

Chronic alcohol use, even in the absence of significant liver disease, is known to alter the intestinal microbiome. The effects of alcohol on the intestinal, hepatobiliary, and immune system physiology, are all postulated to contribute to the observed intestinal dysbiosis. In addition to the hepatotoxic effects of alcohol, the microbiome itself is likely an integral factor for the development and progression of alcohol-associated liver disease.^5^ While there are several studies that have analyzed the compositional changes in the microbiome associated with alcohol-associated liver disease, few have utilized 16S rRNA gene sequencing data from the fecal samples of human subjects. These studies vary widely in methodology, and often grouped patients into a single cirrhosis category, regardless of etiology. Among the studies which purely looked at patients with alcohol-associated liver disease, few used 16S rRNA gene sequencing data of fecal samples from patients with alcoholic hepatitis.^6,8^

Moreover, little is known about how particular microbiome changes are associated with alcoholic hepatitis disease severity or specific clinical parameters. In 2016, Llopis et al. compared the intestinal microbiome of humans with moderate versus severe alcoholic hepatitis prior to transplanting the microbiome into germ-free and conventional mice. This single-center study enrolled a total of 38 patients (22 with alcoholic hepatitis) and classified severity by a histologic scoring system, specifically requiring a neutrophilic infiltrate for severe disease.^7^ Similarly, Smirnova et al. described the microbiome of alcoholic hepatitis by disease severity, but utilized model for end-stage liver disease (MELD) score rather than liver histology to classify severe disease. Patients in this study were enrolled at three centers in the US, 10 and 24 patients were classified as moderate and severe alcoholic hepatitis groups, respectively.^9^

The aim of our study was to characterize the gut microbiome of alcoholic hepatitis patients enrolled in an observational study at multiple centers worldwide.

## Methods

### Patients

The patient cohort has been described before.^8,10,11^ In brief, alcoholic hepatitis patients were enrolled within the InTeam Consortium (ClinicalTrials.gov identifier number: NCT02075918). Seventy-four patients with alcoholic hepatitis hospitalized in nine centers across the United States, Mexico, and Europe, which were enrolled between June 2014 and April 2017, were included in this study. The inclusion criteria for alcoholic hepatitis were as follows: in the last three months prior to enrollment, patients must (1) meet criteria for excessive alcohol use (>50 g/day for men and >40 g/day for women), (2) meet laboratory criteria of aspartate aminotransferase (AST) > alanine aminotransferase (ALT) and total bilirubin >3 g/dl, and (3) have liver biopsy and/or clinical picture consistent with alcoholic hepatitis (all liver biopsies were performed only if part of routine clinical care). Patients carrying diagnoses with other hepatobiliary pathology including autoimmune liver disease (ANA >1/320), chronic viral hepatitis, hepatocellular carcinoma, and complete portal vein thrombosis were excluded. Similarly, patients with other significant comorbidities such as diabetes, inflammatory bowel disease, any terminal condition, or clinically significant cardiac, pulmonary, or renal conditions were excluded. Other exclusion criteria included pregnancy and lack of written informed consent. The protocol was approved by the Ethics Committee of each participating center. Written informed consent was obtained from each subject.

### Bacterial DNA extraction and 16S rRNA sequencing

16S rRNA sequencing of human stool samples was previously described.^8^ Raw 16S sequence reads are available for download in the NCBI Sequence Read Archive (SRA) associated with Bioproject PRJNA525701.^8^ A standardized protocol for fecal sample collection across all centers was used. Further processing of fecal samples was performed at one time concurrently, at one single center.

### Statistical analysis

Results are expressed as median and range unless stated otherwise. The bacterial sequence reads were normalized to get the proportional, relative abundance for further statistical analysis. Two groups were compared using the Mann-Whitney-Wilcoxon rank-sum test for continuous and Fisher’s exact test for categorical variables. Spearman’s correlations were conducted to correlate the relative bacterial abundance at genus level with clinical parameters. Partial Spearman was used to adjust correlations between two numeric variables for cofactors. Multivariate analysis by linear models (MaAsLin), a multivariate statistical framework, was used to detect independent associations between clinical metadata and microbial community abundance.^12^ Only significant associations (*P* < .05) were plotted in the respective graphs. Bray–Curtis dissimilarity matrices were determined for the principal coordinate analysis (PCoA) and *P* values were determined by permutational multivariate analysis of variance (PERMANOVA) while adjusting for potentially confounding factors. Multivariate logistic regression analysis was further used to adjust associations for potentially confounding factors. All statistical tests were two-sided. For all analyses, *P* values of 0.05 or less were considered to be statistically significant. Statistical analysis was performed using R statistical software, R version 3.5.1, 2018 the R Foundation for Statistical Computing.

## Results

The median age of the study cohort was 49  and 67% were male. One-third received steroid treatment, 49% were treated with antibiotics (including prophylactic antibiotics) and the median MELD score was 24 (Table 1). Patients with severe alcoholic hepatitis, stratified based on a MELD score higher than 21, had significantly higher stages of fibrosis, but no significant differences were observed in terms of age, sex, ethnicity, and geographic origin (Supplementary Table 1).

**Table 1.** Characteristics of patients with alcoholic hepatitis (n = 74).

Demographics		
Sex (% male), n (%), n = 73		49 (67.1%)
Age (y), n = 73		49.2 (31.3–74.8)
BMI (kg/m^2^), n = 68		27.1 (16.3–48.3)
Mean Alcohol Intake (g/day), n = 63		104 (113.5*)
Ethnicity, n = 73		
Hispanic, n (%)		13 (17.8)
Non-Hispanic, n (%)		60 (82.2)
Geographic Location, n = 74		
USA, n (%)		43 (58.1)
Mexico, n (%)		6 (8.1)
Europe, n (%)		25 (33.8)
Infections and treatment		
Infections, n (%), n = 57		13 (22.8)
Steroids, n (%), n = 72		22 (30.6)
Pentoxifylline, n (%), n = 70		8 (11.4)
Antibiotics, n (%), n = 72		35 (48.6)
Proton Pump Inhibitor, n (%), n = 37		5 (6.8)
Laboratory parameters		
Creatinine (mg/dL), n = 73		0.8 (0.3–8.1)
Bilirubin (mg/dL), n = 73		14.1 (2.5–38.6)
AST (IU/L), n = 73		136.0 (38.0–456)
ALT (IU/L), n = 73		48.0 (15.0–216.0)
Albumin (g/dL), n = 69		2.4 (1.3–4.1)
INR, n = 72		1.8 (1.0–4.4)
GGT (IU/L), n = 34		242.5 (33.0–3632.0)
Platelet count (10^9^/L), n = 70		126.0 (21.0–447.0)
Liver histology		
Stage of Fibrosis, n (%), n = 41	0/1/2/3/4	2 (4.9)/0 (0.0)/6 (14.6)/8 (19.5)/25 (61.0)
Lobular fibrosis, n (%), n = 40	0/1/2/3	4 (10.0)/6 (15.0)/2 (5.0)/28 (70.0)
Pericellular fibrosis, n (%), n = 40	0/1	9 (22.5)/31 (77.5)
Grade of steatosis, n (%), n = 41	0/1/2/3	0 (0.0)/16 (39.0)/12 (29.3)/13 (31.7)
Mallory bodies, n (%), n = 40	0/1	6 (15.0)/34 (85.0)
Bilirubinostasis, n (%), n = 40	0/1/2/3	14 (35.0)/18 (45.0)/1 (2.5)/7 (17.5)
Ballooning, n (%), n = 40	0/1	27 (37.5)/13 (32.5)
Giant mitochondria, n (%), n = 37	0/1	32 (86.5)/5 (13.5)
PMN infiltration, n (%), n = 41	0/1/2	9 (22.0)/18 (43.9)/14 (34.1)
Inflammatory grade, n (%), n = 41	0/1/2	11 (26.8)/27 (65.9)/3 (7.3)
Clinical scores and outcome		
MELD, median (range), n = 72		23.8 (11.7–43.0)
MELD > 21, n (%)		54 (75)
Child-Pugh stage, n (%), n = 71	A/B/C	1 (1.4)/22 (31.0)/48 (67.6)

Antibiotics include prophylactic antibiotics. Values are presented as median (range or *interquartile range) for continuous variables or number (percentage) for categorical variables. Percentages are calculated based on the actual number of patients in each group where the respective data was available. The number of subjects for which the respective data was available is indicated in the first column. Fibrosis stage, 0 no fibrosis, 1 portal fibrosis, 2 expansive periportal fibrosis, 3 bridging fibrosis, 4 cirrhosis. Lobular fibrosis, 0 no fibrosis, 1 zone 3 (centrilobular) fibrosis, 2 zone 2 + 3 (midzonal) fibrosis, 3 panlobular fibrosis. Pericellular fibrosis, 0 absent, 1 present. Steatosis, 1 mild <33%, 2 moderate <33–66%, 3 marked >66%. Mallory bodies, 0 absent, 1 present. Bilirubinostasis, 0 no, 1 hepato-canalicular, 2 cholangiolar, 3 both. Ballooning, 0 occasional hepatocellular, 1 marked hepatocellular, 2 none present. Megamitochondria, 0 absent, 1 present. PMN infiltration, 0 no, 1 mild, 2 severe. Inflammation, 0 no, 1 mild, 2 severe.

BMI, body mass index; AST, aspartate aminotransferase; ALT, alanine aminotransferase; INR, international-normalized ratio; GGT, gamma-glutamyl transferase; MELD, model for end-stage liver disease; PMN, polymorphonuclear infiltration.

**Figure 1.**
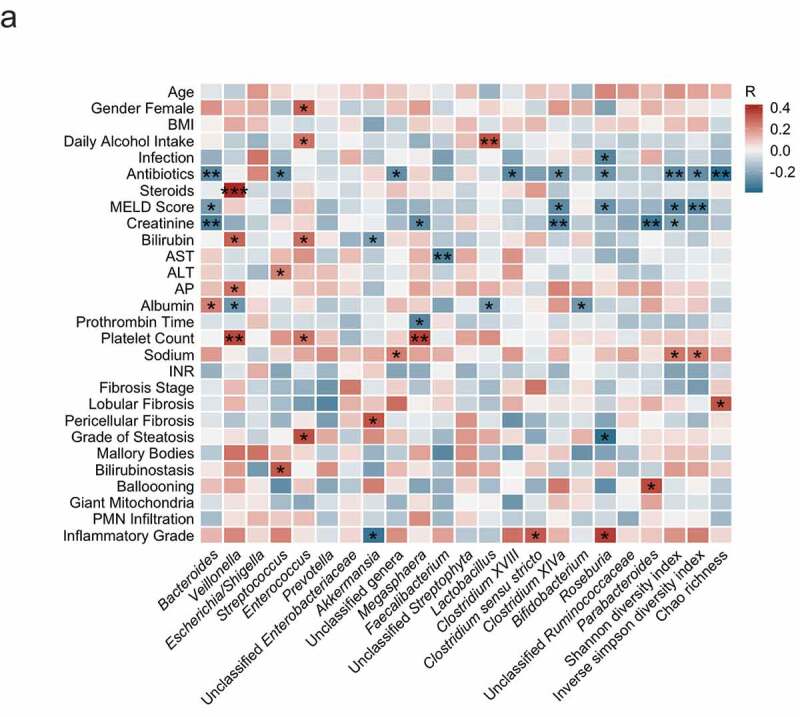
Intestinal bacteria correlate with clinical parameters in alcoholic hepatitis.

To get a first overview about potential associations between the gut bacterial microbiome and clinical, laboratory and histologic parameters in alcoholic hepatitis patients, we performed a heatmap analysis, showing color-coded Spearman correlations between the most abundant gut bacterial taxa at genus level, as well as the overall microbiome diversity with clinical, laboratory, and histological parameters. The most notable associations were related to parameters associated with more disease severity (MELD score, bilirubinemia, albumin levels), treatment modalities (antibiotics and steroids), and inflammatory hepatic changes on liver histology. A higher mean self-reported intake of alcohol was associated with higher abundances of *Enterococcus* and *Lactobacillus* but not with differences in the bacterial diversity (Figure 1).

Heat map representing color-coded Spearman’s correlations of clinical parameters. Red color indicates positive- and blue color negative correlation. All variables are coded from low to high- i.e. red color in antibiotics means antibiotic use is positively correlated with the respective bacterial taxon. *P < .05, *P* > .01, ** *P* < .01, *P* > .001, *** *P* < .001. PMN, polymorphonuclear infiltration; INR, international-normalized ratio; ALT, alanine aminotransferase; AST, aspartate aminotransferase; AP, alkaline phosphatase; BMI, body mass index. MELD, Model for End-stage Liver Disease.

### Severity of alcoholic hepatitis associated with alterations in the composition and diversity of the gut bacterial microbiome

To investigate if liver disease severity in alcoholic hepatitis patients is associated with alterations in the gut bacterial microbiome, we grouped patients according to a MELD score higher than 21, which is a commonly used cutoff to distinguish severe from non-severe alcoholic hepatitis. Since these groups were very uneven in regard to patient numbers (n = 54 in high MELD vs. n = 18 in low MELD), we additionally compared two groups based on the median bilirubin level of 14.1 mg/dl.

**Figure 2.**
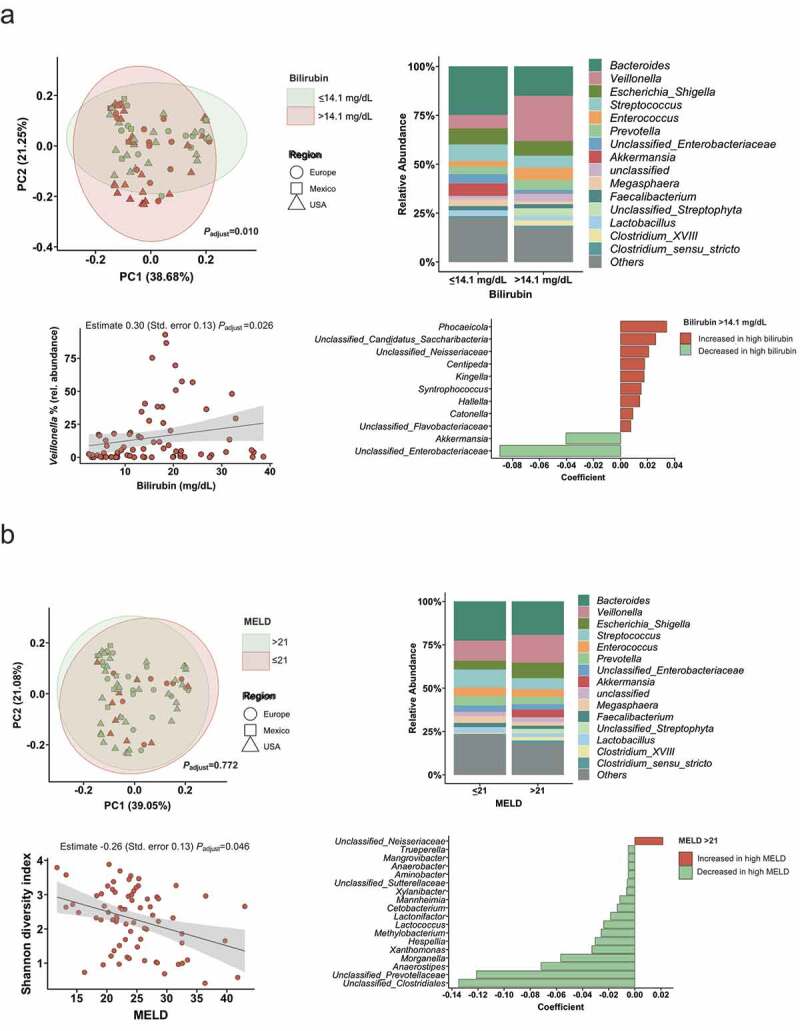
Association of disease severity with the intestinal microbiota.

We compared the overall gut microbiome composition between groups by performing a principal coordinate analysis including 323 gut bacterial taxa at genus level. After adjustment for treatment with antibiotics, steroids and pentoxifylline, ethnicity as well as the region where the patient was enrolled, the high bilirubin patient group (higher than 14.1 mg/dl, n = 36) clustered significantly different (*P*_adjust_ = 0.010) when compared with the lower bilirubin group (less or equal 14.1 mg/dl, n = 37) (Figure 2a, upper left panel). Prominent univariate differences were an increase in *Veillonella* and *Enterococcus*, as well as a reduction in *Akkermansia* in the group with more severe disease (Figure 2a, upper right panel). A higher *Veillonella* abundance was significantly associated with bilirubin levels as continuous variable, independent of antibiotics, steroids, pentoxifylline, ethnicity, and center origin (*P*_adjust_ = 0.026, Figure 2a, lower left panel). MaAsLin analysis revealed significant independent associations between several bacterial taxa and high bilirubin levels, whereas a significantly reduced *Akkermansia* abundance in the high bilirubin group was one major finding (Figure 2a, lower right panel). The Shannon index did not significantly differ among patients with high versus low bilirubin (P_adjust_ = 0.289).

(A) The upper left panel shows the principal coordinate analysis, that was used to show ß-diversity between the groups based on the abundance of 323 bacterial taxa at the genus level. The axes represent the two most discriminating axes using the Bray–Curtis distance metric. The *P* value was determined by permutational multivariate analysis of variance (PERMANOVA). The upper right graph demonstrates the mean relative abundance of sequence reads in each genus for each group (alcoholic hepatitis patients were grouped according to the median bilirubin level of 14.1 mg/dl in the cohort). The lower left panel shows the Spearman correlation between bilirubin levels and the relative abundance of *Veillonella*. The lower right panel shows MaAsLin (Multivariate analysis by linear models), including all 323 bacterial taxa on genus level. Bilirubin >14.1 mg/dl: n = 37; <14.1 or equal: n = 36. (B) The upper left panel shows the principal coordinate analysis, that was used to show ß-diversity between the groups based on the abundance of 323 bacterial taxa at the genus level. Alcoholic hepatitis patients were grouped according to a MELD score higher or lower/equal than 21. The axes represent the two most discriminating axes using the Bray–Curtis distance metric. The *P* value was determined by PERMANOVA. The upper right graph demonstrates the mean relative abundance of sequence reads in each genus for each group. The lower left panel shows the negative Spearman correlation between the Shannon index and the MELD score. The lower right panel shows MaAsLin results. All analyses except for the upper right panels were adjusted for treatment with antibiotics, steroids, pentoxifylline, ethnicity, and the center origin. MELD >21 mg/dl: n = 54; <21 or equal: n = 18.

Only 18 patients had a MELD score lower or equal than 21 compared with 54 patients with a MELD higher than 21. The principal coordinate analysis did not cluster significantly different when comparing the overall microbiome composition between alcoholic hepatitis patients with high versus low MELD (Figure 2b, upper left panel). When we looked into associations between the MELD as continuous with diversity, we found that a higher MELD score was significantly associated with a lower bacterial diversity, as measured by the Shannon index, which was independent of treatment with antibiotics, steroids and pentoxifylline, ethnicity, and the center origin (estimate −0.26, standard error 0.13, *P*_adjust_ = 0.046, Figure 2b, lower left panel). In the MaAsLin analysis, a lower relative abundance of unclassified *Clostridales*, unclassified *Prevotellaceae*, and *Anaerostipes* independently characterized the high MELD patients (Figure 2b, lower right panel).

Overall, these data indicate that a more pronounced disease severity is associated with compositional changes and a reduced bacterial diversity in alcoholic hepatitis patients.

### Gut bacterial alterations associated with antibiotic and steroid treatment

**Figure 3.**
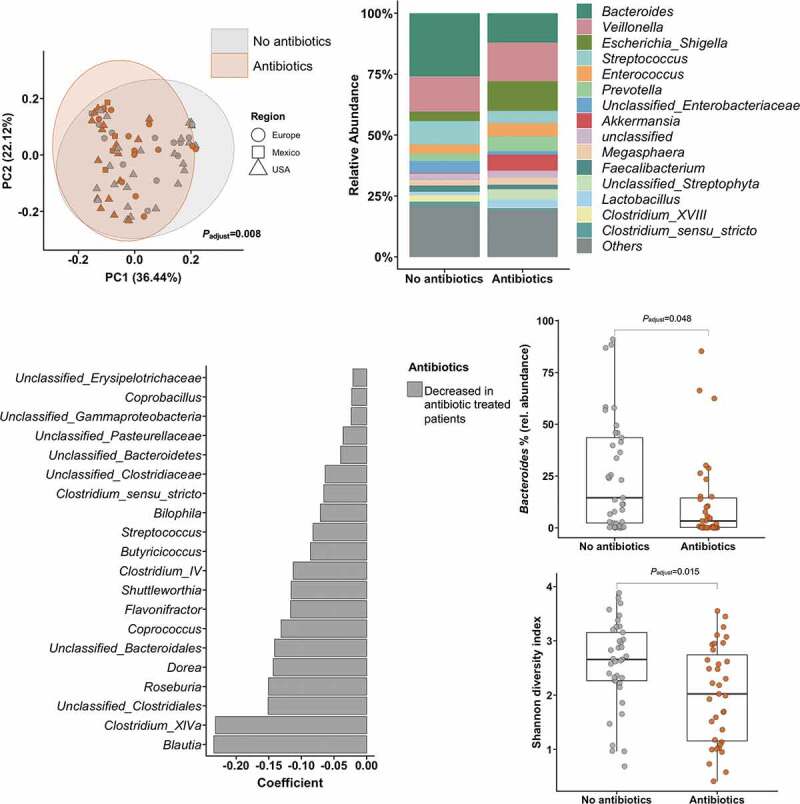
Association of antibiotic treatment with intestinal bacteria.

**Figure 4.**
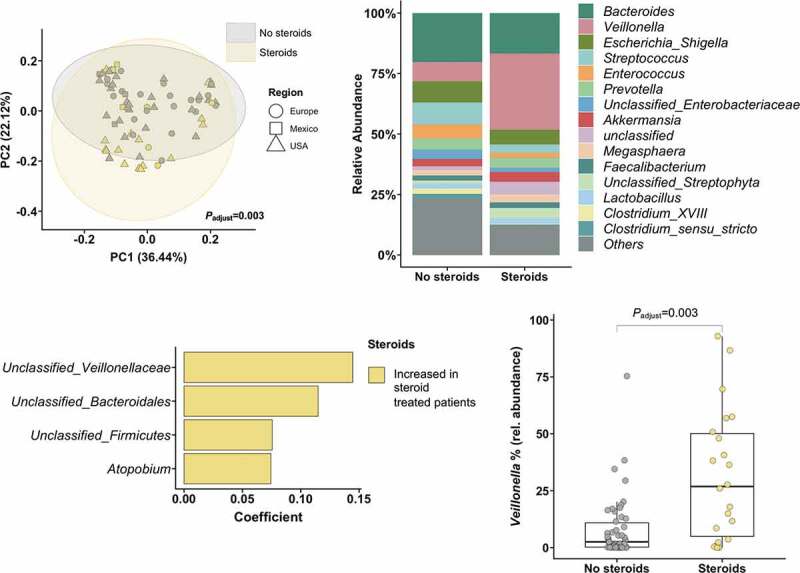
Association of steroid treatment with intestinal bacteria.

Next, we associated treatment with antibiotics and steroids with alterations in the diversity and composition of the gut bacterial microbiome in alcoholic hepatitis patients. The overall gut microbiome composition significantly (*P*_adjust_<0.01) differed among treated groups when performing principal coordinate analyses including the relative abundance of all detected taxa at genus level, adjusted for other treatment (steroids/antibiotics/pentoxifylline respectively), the center origin, levels of bilirubin, albumin, creatinine, the international-normalized ratio (INR), and platelet counts as markers for disease severity (Figures 3 and 4, upper left panel, respectively). When we compared the mean relative abundance of the 15 most abundant taxa at genus level between antibiotic-treated and antibiotic-naïve patients, the most remarkable change was a reduced abundance of *Bacteroides* and an increased abundance of *Escherichia*/*Shigella* in patients treated with antibiotics (mean relative *Bacteroides* abundance: 26% in the antibiotic-naïve group compared with 12% in the antibiotic-treated patient group, *P*_adjust_ = 0.048, Figure 3, upper right and middle right panel). In the MaAsLin analysis, antibiotic use was independently associated with lower levels of *Blautia, Clostridium* spp., *Roseburia, Dorea*, and several other taxa (Figure 3, lower left panel). Further, we observed a reduced bacterial diversity, as measured by the Shannon index (Figure 3, lower right panel, *P*_adjust_ = 0.015).

The upper left panel shows the principal component analysis, that was used to show ß-diversity between the groups based on the abundance of 323 bacterial taxa at the genus level. The axes represent the two most discriminating axes using the jaccard distance metric. The *P* value was determined by the permutational multivariate analysis of variance (PERMANOVA). The upper right graph demonstrates the mean relative abundance of sequence reads in each genus for each group (alcoholic hepatitis patients were grouped according to the treatment with antibiotics, including prophylactic antibiotics (n = 35 (49%)). The middle right panels show the decreased abundance of *Bacteroides* and the decreased Shannon diversity index in patients receiving antibiotics. Wilcoxon-Mann-Whitney test. The lower left panel shows MaAsLin (Multivariate analysis by linear models) results, including all 323 bacterial taxa on genus level. All analyses except for the upper right panel were adjusted for treatment with steroids and pentoxifylline, the center origin, levels of bilirubin, albumin, creatinine, the international-normalized ratio (INR), and platelet counts as markers for disease severity.

The upper left panel shows the principal component analysis, that was used to show ß-diversity between the groups based on the abundance of 323 bacterial taxa at the genus level. The axes represent the two most discriminating axes using the jaccard distance metric. The *P* value was determined by permutational multivariate analysis of variance (PERMANOVA). The upper right graph demonstrates the mean relative abundance of sequence reads in each genus for each group (alcoholic hepatitis patients were grouped according to the treatment with steroids at admission (n = 22 (31%)). The lower right panel shows the increased abundance of *Veillonella* in patients receiving steroids. Wilcoxon-Mann-Whitney test. The lower left panel shows MaAsLin (Multivariate analysis by linear models) results, including all 323 bacterial taxa on genus level. All analyses except for the upper right panel were adjusted for treatment with antibiotics, the center origin, levels of bilirubin, albumin, creatinine, the international-normalized ratio (INR), and platelet counts as markers for disease severity.

In steroid-treated patients, the most remarkable difference compared with patients that did not receive steroids was related to an increased *Veillonella* abundance (mean relative *Veillonella* abundance: 8% in the steroid-naïve group compared with 27% in the steroid-treated patient group, *P*_adjust_ = 0.003, Figure 4, upper and lower right panel), independent of disease severity (levels of bilirubin, albumin, creatinine, INR, and platelet counts), concomitant antibiotic use, ethnicity, or the center origin. The Shannon index did not significantly differ among steroid-treated patients and steroid-naïve patients (*P*_adjust_ = 0.820).

These data indicate that treatment with antibiotics and steroids might lead to alterations in the gut microbiome of alcoholic hepatitis patients.

### Microbiome associations with histologic changes on liver biopsy

**Figure 5.**
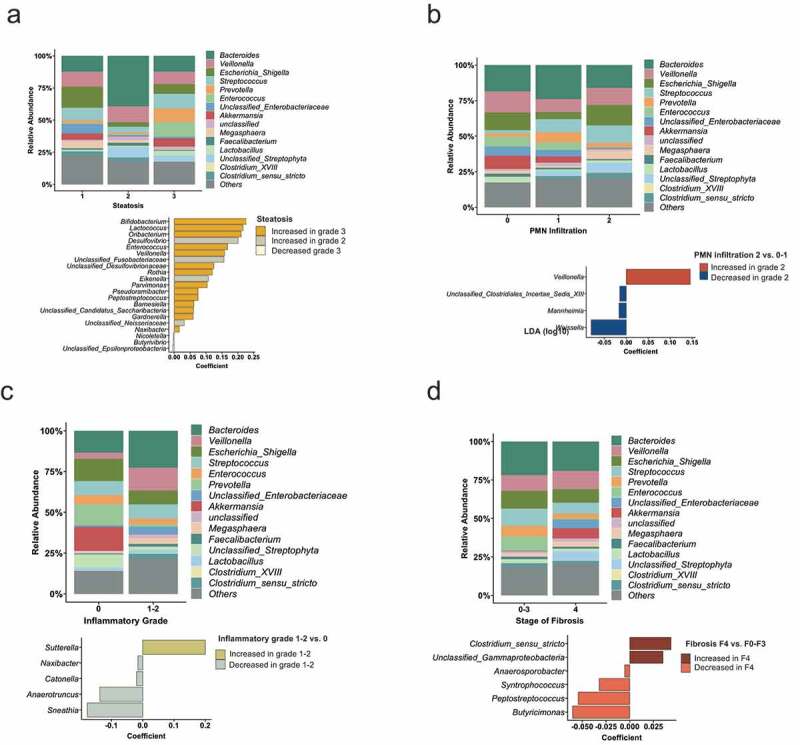
Liver histology features associated with the intestinal microbiota.

We next investigated if liver histology parameters are associated with specific alterations in the composition of the gut bacterial microbiome. Fecal samples from alcoholic hepatitis patients with the highest grade of steatosis contained more *Enterococcus* compared with patients with lower steatosis grades (Figure 5a, upper panel). In the MaAsLin analysis, *Bifidobacterium, Lactococcus, Oribacterium, Desulfovibrio, Enterococcus*, and *Veillonella* were the most increased taxa in patients with grade 3 steatosis (Figure 5a, lower panel). *Akkermansia* was lower abundant in patients with a higher degree of polymorphonuclear infiltration (Figure 5b, upper panel) and a higher degree of histological inflammation (Figure 5c, upper panel). We further observed a higher *Veillonella* abundance in patients with more severe inflammation on liver biopsy (Figure 5c).

(A) Mean relative abundance of sequence reads in each genus for each group (alcoholic hepatitis patients were grouped according to steatosis on liver histology (1, n = 16; 2, n = 12, 3, n = 13)). The lower panel shows MaAsLin (Multivariate analysis by linear models) results, including all 323 bacterial taxa on genus level. (B) Alcoholic hepatitis patients were grouped according to PMN (polymorphonuclear) infiltration on liver histology (1, n = 9; 2, n = 18, 3, n = 14). Mean relative abundance of sequence reads and MaAsLin results, including all 323 bacterial taxa on genus level. (C) Mean relative abundance of sequence reads in each genus for each group (alcoholic hepatitis patients were grouped according to the inflammatory grade on liver histology (0, n = 11; 1–2, n = 30)) and MaAsLin results. (D) Mean relative abundance of sequence reads in each genus for each group (alcoholic hepatitis patients were grouped according to the liver fibrosis stage on liver histology (0–3, n = 16; 4, n = 25)). The lower panel shows MaAsLin results including all 323 bacterial taxa.

Although some lower abundant gut bacterial taxa were associated with less fibrosis, we did not observe major alterations when comparing alcoholic hepatitis patients with cirrhosis (n = 25) versus non-cirrhotic patients (n = 16) (Figure 5d).

## Discussion

This study describes alterations in the fecal microbiota of alcoholic hepatitis patients associated with the severity of the disease, antibiotic and steroid use, and histologic changes on liver biopsy. With increasing data suggesting that the microbiome plays an important role in the pathogenesis of liver disease, there is a need to better characterize particular alterations related to findings commonly encountered in clinical practice.

Prognosis in alcoholic hepatitis is highly variable, but mortality is especially high in severe disease. In the present study, MELD score and degree of hyperbilirubinemia were used as surrogates for disease severity. In patients with more severe liver disease, there was an increased relative abundance *of Veillonella. Veillonella* carries an unknown degree of pathogenicity and has been found to be more prevalent in patients with liver disease.^13,14^ Kakiyama et al. found increased Veillonellaceae at the family level in patients with cirrhosis, and an even more significant increase when the etiology of cirrhosis is alcohol-induced.^14,15^

A diverse variety of microorganisms in the gut are essential for a healthy symbiotic relationship between the microbiome and human host.^5^ Reduced intestinal bacterial diversity has been linked with various negative outcomes, such as obesity and inflammatory bowel disease.^16,17^ While it has been shown that patients with liver cirrhosis tend to have reduced bacterial diversity, there are little data regarding the severity of alcohol-associated liver disease and the heterogeneity of the microbiome.^13^ Ciocan et al. found that patients with severe alcoholic hepatitis had slightly reduced diversity compared to alcoholic controls without liver disease, though not reaching statistical significance. They defined severe disease using a histologic scoring system, although they did not compare with alcoholic hepatitis patients with less severe disease.^6^ In the present study, we found a significant negative correlation between MELD scores and bacterial diversity, independent of antibiotic or steroid treatment. Overall, these findings suggest that severe disease is associated with less bacterial diversity in the microbiome of alcoholic hepatitis patients.

Few studies have described microbiome associations with disease severity in alcoholic hepatitis. A recent study by Smirnova et al. did not find significant differences in the gut microbiota composition or diversity among alcoholic hepatitis patients with moderate versus severe alcoholic hepatitis (defined by MELD of 21 or greater); however, patient numbers were low with only 10 and 24 patients, respectively,^9^ which limits the ability to reach statistical significance. There were some significant differences in individual bacterial taxa between moderate and severe alcoholic hepatitis patients, including an increased abundance of *Actinomycetaceae* and *Fusobacteriaceae*. The authors formulated a microbiome predictive model utilizing MELD score in all alcohol-consuming groups (regardless of the presence of alcoholic hepatitis) and identified the top 20 genera contributing to the model’s ability to accurately predict a MELD score greater than 20. *Veillonellaceae Veillonella, Lachnospiracea incertae sedis, Bacteroidaceae Bacteroides*, and *Streptococcaceae Streptococcus* were the most abundant bacterial taxa in this group.^9^ In our study, we did not see these specific expansions in the high MELD group.

Llopis et al. used a histologic scoring system (alcoholic hepatitis score) to determine alcoholic hepatitis severity and also identified an expansion of *Streptococci*, as well as *Bifidobacteria*, and *Enterobacteria* in patients with severe disease. *Streptococci* and *Enterobacteria* were both positively correlated with the alcoholic hepatitis score, and *Enterobacteria* was also positively correlated with serum bilirubin level.^7^ Lastly, Puri et al. examined the circulating microbiome in patients with alcoholic hepatitis with severity determined by MELD greater than 20. They noted an expansion of *Brevibacterium* and *Staphylococcus* in the circulating microbiome of alcoholic hepatitis patients with severe disease compared to moderate disease. Regression analysis including all alcohol-consuming patients found a negative correlation between MELD score and *Janthinobacterium* and *Enhydrobacter*, two genera of the *Proteobacteria* phylum.^18^ We did not see these associations in our analyses, though it is important to note that the study by Puri et al. uses 16S rRNA gene sequencing from whole blood samples.

Antibiotic use has also been shown to reduce gut bacterial diversity in healthy human subjects.^19^ Also, in our study, antibiotic treatment was associated with a lower bacterial diversity in alcoholic hepatitis patients. The greatest reduction in specific bacterial taxa was seen in genus *Bacteroides*, which is largely considered to be a beneficial organism within the intestinal environment.^20^ While some studies have shown an increased abundance in family Bacteroidaceae amongst cirrhotics,^14,21^ the association appears to invert and show a reduction of Bacteroidaceae when the analysis includes or is limited to alcoholic cirrhosis.^13,15,22^ The antibiotic-treated group in our study also had a significant increase in anaerobes *Bilophila* and *Flavonifractor*, both of which are rare clinical pathogens, but have been associated with systemic inflammation and/or colorectal malignancy.^23,26^ However, whether antibiotic use is causatively linked to these observations, can not be answered with our study design.

We found a marked increase in the relative abundance of *Veillonella* in alcoholic hepatitis patients that had been treated with steroids. In clinical practice, steroid use is generally reserved for severe alcoholic hepatitis, often defined by Maddrey’s Discriminant Function (DF) Score exceeding 32 and if no contraindications are present. Since we had already identified *Veillonella* to be more prevalent in patients with severe hyperbilirubinemia (which is a component of the Maddrey’s DF score), we deduced that at least some of this association was likely attributable to disease severity. However, the association between a higher *Veillonella* abundance and steroid use remained significant after performing a multivariate logistic regression analysis controlling for parameters associated with disease severity (including bilirubin level). While further mechanistic studies are needed to elucidate the potential pathogenic function of *Veillonella* in alcoholic hepatitis, this study corroborates that *Veillonella* is also positively correlated with disease severity and may be independently expanded by the use of steroids.

The fecal microbiota in alcoholic patients were also evaluated for associations with various liver histologic changes. Severe steatosis is associated with an increase in genera *Enterococcus*, which are largely regarded as pathologic organisms. When we evaluated inflammatory histologic changes, such as PMN infiltration and inflammatory grade, *Akkermansia* abundance was inversely correlated with increased inflammation and also reduced in patients with higher bilirubin levels. *Akkermansia* is a well-described beneficial inhabitant of the human microbiome and is important for fatty acid metabolism. Its depletion has been implicated in obesity and fatty liver disease, and supplementation of *Akkermansia* has even been shown to decrease serum triglyceride and alanine aminotransferase levels in obese mice.^27^ Recently, Addolorato et al. published their findings of significant *Akkermansia* reduction in patients with alcohol use disorder, irrespective of the degree of liver disease.^28^ In line, Grander et al. found a depletion of *Akkermansia* in fecal samples from alcoholic hepatitis patients and *Akkermansia* supplementation protected mice against liver injury in experimental alcoholic liver disease.^29^ Our study suggests that the reduction in *Akkermansia* abundance is related to the degree of liver inflammation as seen on liver biopsy in patients with alcoholic hepatitis.

The main strength of our study is its international multicenter design, which aims to control for various geographical and ethnic factors that may affect microbiome composition. To our knowledge, our study sequenced fecal samples from the largest number of alcoholic hepatitis patients, that were also enrolled in multiple centers including Europe, the United States, and Mexico. However, our study is descriptive and limited by its inability to determine causation. Further, the use of medications other than antibiotics, steroids, and pentoxifylline was incompletely reported; therefore, we can not rule out that other concomitant medication use might have influenced our results. Further mechanistic studies are required to better understand the role of the microbiome in the pathogenesis and progression of alcoholic hepatitis.

In summary, we observed compositional alterations, such an increased *Veillonella* abundance, and a lower bacterial diversity among alcoholic hepatitis patients with more severe disease. We further observed a reduction in *Bacteroides* in antibiotic-treated patients and an enrichment of *Veillonella* in patients treated with steroids. The gut bacterial microbiome might be an attractive target to prevent and treat this deadly disease.

## Supplementary Material

Supplemental Material
