# Supplementary material for: Changes in the fecal bacterial microbiota associated with disease severity in alcoholic hepatitis patients
Source: Gut Microbes. 2020 Jul 20;12(1):1785251. doi: 10.1080/19490976.2020.1785251 (PMC7524371; doi:10.1080/19490976.2020.1785251)
Supplement: Supplemental Material [file KGMI_A_1785251_SM0985.docx]

**Table 1:** Characteristics of patients with alcoholic hepatitis (n=74)

| Demographics |  |  |
| --- | --- | --- |
| Sex (% male), n (%), n=73 |  | 49 (67.1%) |
| Age (years), n=73 |  | 49.2 (31.3-74.8) |
| BMI (kg/m^2^), n=68 |  | 27.1 (16.3-48.3) |
| Mean Alcohol Intake (g/day), n=63 |  | 104 (16.8-1750) |
| Ethnicity, n =73 |  |  |
| Hispanic, n (%) |  | 13 (17.8) |
| Non-Hispanic, n (%) |  | 60 (82.2) |
| Geographic Location, n=74 |  |  |
| USA, n (%) |  | 43 (58.1) |
| Mexico, n (%) |  | 6 (8.1) |
| Europe, n (%) |  | 25 (33.8) |
|  |  |  |
| Infections and treatment |  |  |
| Infections, n (%), n=57 |  | 13 (22.8) |
| Steroids, n (%), n=72 |  | 22 (30.6) |
| Pentoxifylline, n (%), n=70 |  | 8 (11.4) |
| Antibiotics, n (%), n=72 |  | 35 (48.6) |
| Proton Pump Inhibitor, n (%), n=37 |  | 5 (6.8) |
|  |  |  |
| Laboratory parameters |  |  |
| Creatinine (mg/dL), n=73 |  | 0.8 (0.3-8.1) |
| Bilirubin (mg/dL), n=73 |  | 14.1 (2.5-38.6) |
| AST (IU/L), n=73 |  | 136.0 (38.0-456) |
| ALT (IU/L), n=73 |  | 48.0 (15.0-216.0) |
| Albumin (g/dL), n=69 |  | 2.4 (1.3-4.1) |
| INR, n=72 |  | 1.8 (1.0-4.4) |
| GGT (IU/L), n=34 |  | 242.5 (33.0-3632.0) |
| Platelet count (10^9^/L), n=70 |  | 126.0 (21.0-447.0) |
|  |  |  |
| Liver histology |  |  |
| Stage of Fibrosis, n (%), n=41 | 0 / 1 / 2 / 3 / 4 | 2 (4.9) / 0 (0.0) / 6 (14.6) / 8 (19.5) / 25 (61.0) |
| Lobular fibrosis, n (%), n=40 | 0 / 1 / 2 / 3 | 4 (10.0) / 6 (15.0) / 2 (5.0) / 28 (70.0) |
| Pericellular fibrosis, n (%), n=40 | 0 / 1 | 9 (22.5) / 31 (77.5) |
| Grade of steatosis, n (%), n=41 | 0 / 1 / 2 / 3 | 0 (0.0) / 16 (39.0) / 12 (29.3) / 13 (31.7) |
| Mallory bodies, n (%), n=40 | 0 / 1 | 6 (15.0) / 34 (85.0) |
| Bilirubinostasis, n (%), n=40 | 0 / 1 / 2 / 3 | 14 (35.0) / 18 (45.0) / 1 (2.5) / 7 (17.5) |
| Ballooning, n (%), n=40 | 0 / 1 | 27 (37.5) / 13 (32.5) |
| Giant mitochondria, n (%), n=37 | 0 / 1 | 32 (86.5) / 5 (13.5) |
| PMN infiltration, n (%), n=41 | 0 / 1 / 2 | 9 (22.0) / 18 (43.9) / 14 (34.1) |
| Inflammatory grade, n (%), n=41 | 0 / 1 / 2 | 11 (26.8) / 27 (65.9) / 3 (7.3) |
|  |  |  |
| Clinical scores and outcome |  |  |
| MELD, median (range), n=72 |  | 23.8 (11.7-43.0) |
| MELD > 21, n (%) |  | 54 (75) |
| Child-Pugh stage, n (%), n=71 | A / B / C | 1 (1.4) / 22 (31.0) / 48 (67.6) |

Antibiotics include prophylactic antibiotics. Values are presented as median (range) for continuous variables or number (percentage) for categorical variables. Percentages are calculated based on the actual number of patients in each group where the respective data was available. The number of subjects for which the respective data was available is indicated in the first column. Fibrosis stage, 0 no fibrosis, 1 portal fibrosis, 2 expansive periportal fibrosis, 3 bridging fibrosis, 4 cirrhosis. Lobular fibrosis, 0 no fibrosis, 1 zone 3 (centrilobular) fibrosis, 2 zone 2+3 (midzonal) fibrosis, 3 panlobular fibrosis. Pericellular fibrosis, 0 absent, 1 present. Steatosis, 1 mild < 33%, 2 moderate < 33-66%, 3 marked > 66%. Mallory bodies, 0 absent, 1 present. Bilirubinostasis, 0 no, 1 hepato-canalicular, 2 cholangiolar, 3 both. Ballooning, 0 occasional hepatocellular, 1 marked hepatocellular, 2 none present. Megamitochondria, 0 absent, 1 present. PMN infiltration, 0 no, 1 mild, 2 severe. Inflammation, 0 no, 1 mild, 2 severe.

BMI, body mass index; AST, aspartate aminotransferase; ALT, alanine aminotransferase; INR, international normalized ratio; GGT, gamma-glutamyl transferase; MELD, model for end-stage liver disease; PMN, polymorphonuclear infiltration.
